# Growth and physiological changes in continuously cropped eggplant (*Solanum melongena* L.) upon relay intercropping with garlic (*Allium sativum* L.)

**DOI:** 10.3389/fpls.2015.00262

**Published:** 2015-04-24

**Authors:** Mengyi Wang, Cuinan Wu, Zhihui Cheng, Huanwen Meng

**Affiliations:** College of Horticulture, Northwest A&F UniversityYangling, China

**Keywords:** malondialdehyde, superoxide dismutase, peroxidase, polyphenol oxidase, phenylalanine ammonia-lyase, growth, yield

## Abstract

Relay intercropping represents an alternative for sustainable production of vegetables, but the changes of internally antioxidant defense combined with the growth and yield are not clear. Field experiment was carried out to investigate the malondialdehyde (MDA) content and activity levels of superoxide dismutase (SOD), peroxidase (POD), polyphenol oxidase (PPO), and phenylalanine ammonia-lyase (PAL) in eggplant (*Solanum melongena* L.) and plant height, stem diameter, maximal leaf area, and yield of eggplant grown under successive cropping in the year 2011 and 2012 to see if relay intercropping with garlic (*Allium sativum* L.) could benefit to eggplant growth and yield. Three experimental treatments with three repeats in each were carried out (completely randomized block design): eggplant monoculture (CK), eggplant relay intercropping with normal garlic (NG), and eggplant relay intercropping with green garlic (GG). In both years, the MDA content was significantly lower and SOD and POD activities were generally lower in NG and GG compared with CK in most sampling dates. PPO activity trends were generally opposite to those of POD. The general trend of PAL activity was similar to MDA. The plant height and stem of eggplant was lower, but the maximal leaf area was larger in NG and GG in 2011; in 2012 the plant growth was stronger in relay intercropping treatments. For eggplant yield in 2011, NG was 2.85% higher than CK; after the time for the green garlic pulled out was moved forward in 2012, the yield was increased by 6.26 and 7.80%, respectively, in NG and GG. The lower MDA content and enzyme activities in relay intercropping treatments showed that the eggplant suffered less damage from environment and continuous cropping obstacles, which promoted healthier plant. Thus from both the growth and physiological perspective, it was concluded that eggplant/garlic relay intercropping is a beneficial cultivation practice maintaining stronger plant growth and higher yield.

## Introduction

Continuous cropping is a simple, repetitive agronomic practice that is often performed for vegetables cultivated under protection in China due to concerns of land-use efficiency and the cultivation habits of farmers (Xiao et al., 2012). Eggplant (*Solanum melongena* L.) is one of the most popular vegetables worldwide and is grown in most parts of the world. China, which is currently the largest producer of eggplant, has experienced a rapid increase in areas of protected cultivation. However, the continuous cropping of eggplant, which commonly occurs in China, can have negative effects, including increase in autotoxins in the plant which blocks its growth; decrease in resistance to bad environment; slow development; and reduce in yield and quality.

One beneficial agricultural measure that alleviates such continuous cropping obstacles is crop rotation, which is the practice of sequentially growing different crops in the same field. Crop rotation is commonly practiced to decrease the incidence of soil-borne pathogens and to maintain soil fertility (Hiddink et al., 2010). However, as crop rotation rarely occurs in protected plastic tunnel systems because of the limited land area available under the plastic tunnels and Chinese farmers are accustomed to repeatedly planting the same crop in the same field, the cultivation pattern of relay intercropping has recently garnered increasing attention. Relay intercropping in mixed-cropping systems is defined as the overlapping cultivation of two or more crops in the same field, with the second crop planted when the first crop has reached its reproductive stage but has not yet been harvested (Hiddink et al., 2010). Relay intercropping is considered a practical application for basic ecological principles such as diversity, competition, and facilitation (Hauggaard-Nielsen et al., 2008), and this practice has shown enormous potential for improving soil nutrient efficiency and reducing the occurrence of plant pests, weeds, and soil-borne diseases. Especially, compared with corresponding sole crops, yield advantages have been recorded in many intercropping systems, including wheat/maize (Li et al., 2001), barley and annual medic (Sadeghpour et al., 2014), Chinese cabbages/garlic (Unlu et al., 2010), etc. Moreover, Famaye et al. (2011) found that the plant height and leaf area vegetative growth were significantly higher in the intercrops than the sole cocoa but not significantly different in all the months. Plant vigor was significantly higher in cocoa/plantain intercrop. Another study found that tomato plants intercropped with marigold or pigweed grew longer stems (26–33%) and thinner leaves (33–35%) than plants grown alone (Gómez-Rodríguez et al., 2007).

To maximize the effectiveness of relay intercropping, the correct combination of plants must be selected. Certain crop species, such as garlic (*Allium sativum* L.), onion (*A. cepa* L.), and welsh onion (*A. fistulosum* L.), have strong flavors that can repel certain pests and pathogens. Garlic is an important plant that is used worldwide as a flavoring and cover crop (Han et al., 2012), and its root exudates and shoot volatiles have a natural broad-spectrum antimicrobial activity that enables this crop to protect adjacent crops from pest attacks and pathogen infection while providing negligible competition for light and water (Cheng et al., 2007). Researchers have also found that garlic root exudates have a significant inhibitory effect on *Phytophthora capsici* (Khan and Cheng, 2010; Khan et al., 2011).

Plants may encounter a variety of external stresses during growth, including water and salt stress and extreme temperatures. For crops under continuous cropping, additional stress arises from continuous cropping obstacles, which include the frequent occurrence of pests, the gradual accumulation of serious pathogens, decline of soil physicochemical properties, and accumulation of certain poisonous root exudates in the soil (Chen et al., 2011b). All of these stresses threaten plant growth and may cause the plant to produce reactive oxygen species (ROS), which act as early signals of a plant’s defense response to external stress and serve as secondary messengers for subsequent defense reactions (Asada, 2006; Miller et al., 2007; Möller and Sweetlove, 2010; Wrzaczek et al., 2013; Yin et al., 2015), they can cause serious injury, including lipid peroxidation, membrane destruction, protein denaturation, and DNA mutation (Mittler, 2002; Davey et al., 2005; Yamauchi et al., 2008; Çelekli et al., 2013; Suleman et al., 2013).

Malondialdehyde (MDA) is a decomposition product of the polyunsaturated fatty acid hydroperoxides that are generated from reactions with ROS (Suzuki and Mittler, 2006; Tommasino et al., 2012). MDA is a reactive aldehyde, and its production may be used as a biomarker to measure the level of oxidative stress in an organism (Davey et al., 2005; Rio et al., 2005). MDA bonds with molecules such as proteins, nucleic acids, and amino acids to form insoluble compounds that can disturb cell processes and influence a plant’s normal growth and development (Huang et al., 2006).

Various external stresses often induce the activity of free radical detoxification enzymes in plants (Tang et al., 2010; Rai et al., 2012). Superoxide dismutase (SOD) (EC 1.15.1.1), an important antioxidant defense enzyme and a major scavenger of O^2-^, catalyzes the dismutation of superoxide radical anions into O_2_ and H_2_O_2_ (Huseynova et al., 2014; Shafi et al., 2015). The highly toxic H_2_O_2_ is then scavenged by catalase (CAT) (EC 1.11.1.6) and peroxidase (POD) (EC 1.11.1.7). POD degrades H_2_O_2_ through the oxidation of cosubstrates such as phenolic compounds and/or antioxidants, thereby eliminating the deleterious effects of H_2_O_2_ on plants (Asada, 2006). POD activity is highly correlated with a wide range of plant physiological processes (Hiraga et al., 2001; Passardi et al., 2005; Kravic et al., 2013), including the construction and eventual lignification of cell walls, resistance to insects and pathogens, and wound healing (Moore et al., 2003; Almagro et al., 2009). Polyphenol oxidase (PPO) (monophenol, *o*-diphenol: EC 1.14.18.1, EC 1.10.3.2), another widely distributed resistance-related enzyme, is a copper-containing oxidoreductase that catalyzes two distinct reactions involving phenolic compounds and molecular oxygen. The proposed physiological roles of PPO in higher plants include producing the browning response (Richter et al., 2012), scavenging molecular oxygen in chloroplasts, participating in the plant’s defense system, wound-induced rooting, and wound healing (Ramiro et al., 2006; Constabel and Barbehenn, 2008; Richter et al., 2012). Both POD and PPO are important enzymes that are responsible for the oxidation of phenolic compounds (Takahama, 2004; Doorn and Ketsa, 2014). In addition to that caused by PPO, enzymatic browning may result from the oxidation of phenols initiated by phenylalanine ammonia-lyase (PAL) (EC 4.3.1.5), as PAL activity in wounded tissues produces phenolic compounds that are responsible for tissue browning (Soliva-Fortuny and Martín-Belloso, 2003). PAL generally occurs at low levels in normal tissues, though its activity greatly increases upon infection and stress (Ritter and Schulz, 2004; Godinez-Vidal et al., 2008; Wada et al., 2014).

Many reports have discussed the ability of relay intercropping systems to reduce soil-borne disease and increase soil fertility (Li et al., 2005; Hiddink et al., 2010; Xiao et al., 2012). However, little information is available regarding the combined effects of relay intercropping with normal garlic or green garlic on the resistance-related enzymes and the growth and yield of eggplant. Therefore, this study examines the MDA content and activities of resistance-related enzymes and the plant height, stem diameter, maximal leaf area, and yield of eggplant to determine whether relay intercropping with garlic can lead to benefits from comprehensively growth and physiological perspectives.

## Materials and Methods

### Experimental Site

The experiment was conducted from March 2011 to November 2012 in a plastic tunnel at Horticultural Experimental Station (34°17^′^ N, 108°04^′^ E) of Northwest A&F University, Yangling, Shaanxi Province, China. The annual average temperature at the study site is 12.9°C, with extreme temperatures of 38°C and -11°C, and the frost-free period is over 200 days. Under plastic tunnels, the temperature showed parabola-like trend in both 2011 and 2012 whether the maximum or minimum temperature, and the highest temperature can reach approximately 50°C in Summer, and the lowest temperature is approximately -10°C in Winter.

The soil characteristic at the experimental location, which is a brown, loamy, alkaline Orthic Anthrosol, was described in our previous paper (Wang et al., 2014). The pH of the soil is 7.8 (1:1 water), and it contains 27.02 g organic matter, 1.38 g total nitrogen, 0.96 g total phosphorus, and 14.31 g total potassium per kilogram of dry soil. In the 0–20 cm soil layer before eggplant transplantation, the ammonium nitrogen concentration was 57.17 mg⋅kg^-1^, the available phosphorus was 57.65 mg⋅kg^-1^, and the exchangeable potassium was 224.01 mg⋅kg^-1^.

### Experimental Design

Eggplant (*S melongena* L.) was relay intercropped as the main crop with normal garlic (NG, sowing cloves of cv. G110 in September and harvesting garlic bulbs in the next April in both years) or green garlic (GG, sowing whole bulbs of cv. G064 in July or August and harvesting green garlic three or four times within the 3 months after planting and after the green garlic had grown to approximately 30 cm high). The green garlic was pulled out in April in 2011 but in late March in 2012 which was the same time as eggplant transplanting because the negative effect of sunlight blocking from thick green garlic on the eggplant seedlings was found in 2011, and the time of pulling out green garlic was moved forward in 2012. Eggplant monoculture (CK) was used as the control. All the treatments were the same with our previous study (Wang et al., 2014).

The experiment was a completely randomized block design with three replications. Each plot contained two beds of 1.2 m × 3.5 m. In both the monoculture and relay intercropping treatments, the eggplant plants were spaced 50 cm apart, the rows were spaced 80 cm apart, and there were two rows per bed and seven plants per row. In the middle of each bed, between the two rows of eggplant, three rows of garlic cloves were planted in the NG treatment (20 cm row spacing and 6 cm plant spacing, with 141 cloves for each bed), with four rows of garlic bulbs planted in the GG treatment (12 cm row spacing and adjacent in each row, with 8.48 kg bulbs for each bed). Eggplant was grown in this field for three successive years: the plants were transplanted on March 19, 2010, March 22, 2011, and March 24, 2012, and uprooted on November 25 in all three years. In 2010, the seed cloves and bulbs were planted on September 15, and the three treatments were established. The bulbs were planted on August 1, 2011 and July 20, 2012, and the cloves were planted on September 15 in both years.

Prior to eggplant transplantation, the experimental field was plowed and fertilized with 1.5 kg “PengDiXin” (organic matter content ≥30%, N+P_2_O+K_2_O content ≥4%, humic acid content ≥20%, trace element content ≥2%, organic sylvite content ≥5%; Zhengzhou Jinzheng Bio-chemical Co., LTD, Henan Province, China), 0.15 kg double superphosphate (total P content ≥46%, available P content ≥44%), and 0.15 kg “SaKeFu” compound fertilizer (total primary nutrient content ≥40%; Sino-Arab Chemical Fertilizers Co., LED, Hebei Province, China) per bed. Farming management was performed following local conventions. A topdressing (a complete fertilizer called “JinBa” with humic acid content ≥3%, trace element content ≥6%, N+K_2_O content ≥18%, and phosphate and K-solubilizing agent content ≥5%; Rishengjiufeng Biotechnology Co., LTD, Beijing, China) and irrigation were applied to each bed according to local farming conventions in both the eggplant-only period and the relay intercropping period. Irrigation but no topdressing was applied as needed for the normal garlic or green garlic during their sole cultivation (Wang et al., 2014). For eggplant, vine tying, pruning, and other farm management were administered following local conventions. During the appropriate growth stages, the eggplant plants were double-pole trained, and the vine branches were suspended from nylon ropes.

### Physiological Measurements

#### Leaf Sampling

Leaf samples were randomly collected between 10:00 and 10:30 a.m. from fully expanded leaves in the upper-middle portions of the eggplant plants, collecting one leaf per plant from six plants per treatment. Disease- and pest-free leaves were removed from the base, without the petiole. The first sampling per stubble was at 36/24 days after eggplant transplantation on April 28/17 in 2011 and 2012, respectively. Additional samples were taken on May 18/June 17 (full eggplant production after the garlic harvest), July 25/15 (5 days before planting green garlic), August 30/September 10 (15/5 days before planting normal garlic), October 9 (eggplant/garlic relay intercropping period), and October 30/20 (the later growth stage of eggplant) in 2011 and 2012, respectively.

Each leaf sample was placed in a plastic bag and then placed in crushed ice in a foam box immediately after collected. The surfaces of the leaves were washed with tap water and distilled water and then gently dried with absorbent paper. Afterward, the leaves were cut into pieces (omitting thick veins), packaged in aluminum foil, frozen in liquid nitrogen, and placed in a -80°C ultralow temperature freezer. All of the study parameters were subsequently measured as soon as possible.

#### MDA and Enzyme Crude Extract

The crude extract for MDA and resistance-related enzyme, including SOD, POD, PPO, and PAL, was prepared using the methods described by Gao (2006), with some modifications. Leaf samples (0.500 g) were ground with 2 mL of cold extraction buffer (0.05 M phosphate buffer, pH 7.8), and the entire mixture was transferred to centrifuge tubes with another 6 mL of the same extraction buffer and centrifuged for 20 min at 10,000×*g* and 4°C. The supernatant was used to determine the content of MDA and enzyme activities for each treatment; the measurements were performed in triplicate.

#### Determination of MDA Content and SOD, POD, PPO, and PAL Activities

The MDA content was measured using the thiobarbituric acid (TBA) reaction (Zhang, 2004). Two milliliter of the extract supernatant was mixed with 2 mL 0.6% (w/v) TBA solution dissolved in 5% (v/v) trichloroacetic acid (TCA), heated in boiling water for 10 min, and then cooled to allow the flocculate to sediment. The supernatant was used for the spectrophotometric determination of MDA. The absorbance at the wavelength of 450 and 532 nm was measured and subtracted from the absorbance at 600 nm. The MDA content was expressed as the amount of substance per gram of fresh leaves (nmol⋅g^-1^Fw).

Total SOD activity was estimated by the inhibition of the photochemical reduction of nitro blue tetrazolium (NBT) (Gao, 2006). The reaction mixture contained 1.5 mL 0.05 M phosphate buffer (pH 7.8), 0.3 mL 0.1 mmol⋅L^-1^ EDTA-Na_2_, 0.3 mL 0.13 mol⋅L^-1^ methionine, 0.3 mL 0.75 mmol⋅L^-1^ NBT, 0.3 mL 0.02 mmol⋅L^-1^ riboflavin, 0.05 mL enzymatic extract, and 0.25 mL distilled water in a total volume of 3 mL for the reaction mixture. After exposure to fluorescent light (86.86 μmol⋅m^-2^⋅s^-1^) for 10–20 min (endpoint determined by the color of the reaction solution), the absorbance was recorded at the wavelength of 560 nm. SOD activity was determined as 50% inhibition of the NBT reduction caused by the superoxides generated from the reaction of photo-reduced riboflavin and oxygen. The total SOD activity was expressed in units per gram of fresh leaves (u⋅g^-1^Fw).

The guaiacol method was used for the determination of POD activity (Charles et al., 1998). A reaction mixture was prepared using 50 mL 0.05 M phosphate buffer (pH 7.8), 28 μL guaiacol, and 19 μL 30% H_2_O_2_ (v/v); 3.5 mL of the reaction mixture solution was placed into a cuvette with a 1 cm path length. The increase in absorbance at the wavelength of 470 nm was recorded over 3 min at 30 s intervals after the addition of 0.5 mL enzyme extract. The results were presented as D_470_ per minute per gram of fresh leaves (U⋅g^-1^⋅min^-1^).

PPO activity was measured spectrophotometrically by the increase in colored oxidation products within the first 3 min of the reaction (Zheng et al., 2007). After heating at 37°C for 10 min, a mixed solution of 1.5 mL 0.05 M phosphate buffer (pH 7.8) and 1.0 mL 0.1 mol⋅L^-1^ catechol was placed into a cuvette with a 1 cm path length. Immediately after the addition of 0.5 mL enzyme extract to initiate the reaction, the enzyme activity was measured at the wavelength of 410 nm every 30 s for 3 min. One unit of PPO was defined as the amount of enzyme that produced a change in absorbance of 0.001 min^-1^ (0.001ΔA⋅min^-1^).

PAL activity was assayed using the procedure developed by Gao (2006). The enzyme activity was measured in a mixture (4 mL) containing 2.7 mL 0.05 M phosphate buffer (pH 7.8), 1 mL 0.02 mol⋅L^-1^ L-phenylalanine, and 0.3 mL enzyme extract at 30°C for 60 min; and the reaction was terminated by the addition of 0.2 mL 6 mol⋅L^-1^ HCl. The final mixture was spectrophotometrically measured in a quartz cuvette at the wavelength of 290 nm, and a unit of PAL was defined as the amount needed to produce a change in absorbance of 0.01 per hour at the wavelength of 290 nm, which was equivalent to a 1 mL reaction solution forming 1 μg *trans*-cinnamic acid (A_290_⋅g^-1^⋅h^-1^).

### Eggplant Growth and Yield Record

The morphological parameters were measured on June 20, 2011 and on June 17, 2012 which were in the most vigorous growth periods for characterizing the plant growth. Plant height was measured using tape (0.1 cm) and the stem diameter using electronic vernier caliper (0.01 mm). The maximal leaf area is represented by leaf length multiply leaf width (cm^2^) of the maximal leaf. The plant height is the vertical distance from the leaf tip to the ground under natural conditions; stem diameter is measured at the widest part of the internode above cotyledons. The leaf length is measured from petiole base to blade tip and the leaf width is the extreme breadth of the maximal leaf.

Eggplant yield was recorded as the total harvest of edible mature fruits in 2011 and 2012 and presented as average per plot in kilogram per hectare (kg⋅ha^-1^).

### Statistical Analyses

The data of MDA content and plant enzymes obtained in this study were subjected to an analysis of variance (ANOVA), and all significant differences in physiological parameters among the monoculture and relay intercropping systems were examined according to Fisher’s least significant difference (LSD) test at *P* < 0.05. LSD tests were calculated using PASW Statistics 18.0 software (IBM, Armonk, NY, USA).

## Results

### Effect of Relay Intercropping with Normal Garlic or Green Garlic on the MDA Content in Eggplant Leaves

**FIGURE 1:**
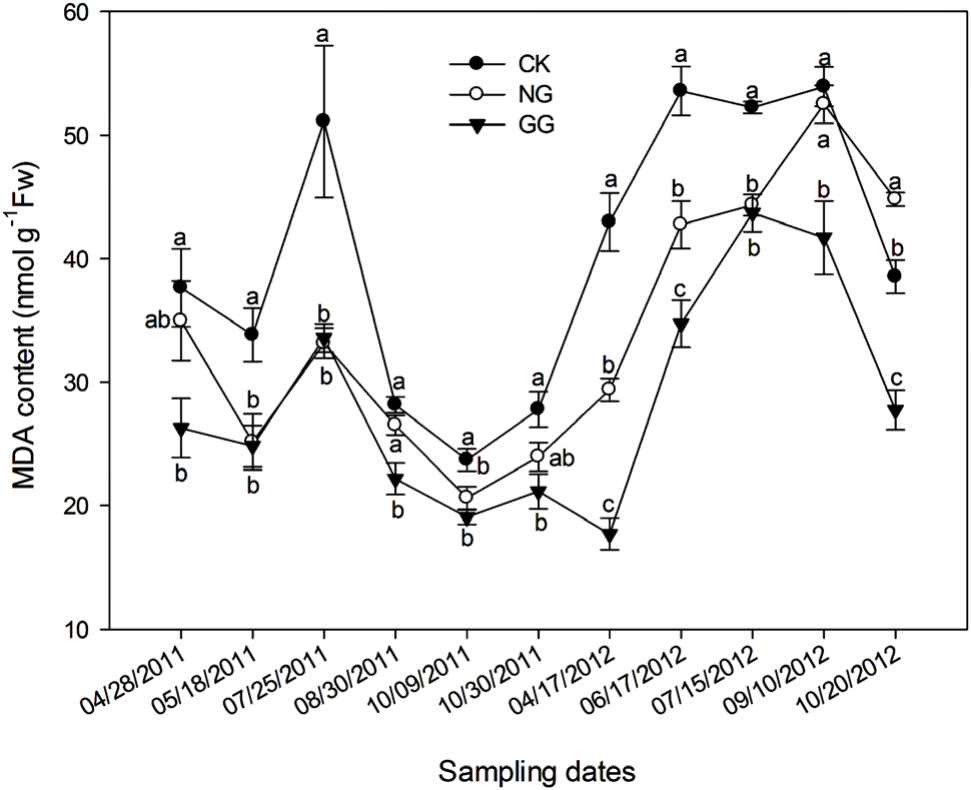
**Effect of relay intercropping with normal garlic or green garlic on the malondialdehyde (MDA) content in eggplant leaves.** Error bars represent the SE of the mean. The letters a, b, and c indicate significant differences at the *p* < 0.05 level (ANOVA and LSD); *n* = 3. CK, eggplant monoculture; NG,eggplant under relay intercropping with normal garlic; GG, eggplant under relay intercropping with green garlic.

The overall trend of the MDA content was consistent in both 2011 and 2012: an initial increase that peaked at the blooming and fruit-bearing stages and subsequently decreased (**Figure 1**), which was consistent with the seasonal changes in temperature. In both years of the experiment, the MDA content in the NG treatment was lower than that in the CK treatment on most sampling dates, with the exception of October 20, 2012. In addition, for all sampling dates, the MDA content was significantly lower in the GG treatment than in the CK treatment. The MDA content in the GG treatment was also lower than that in the NG treatment for most sampling dates, with the exception of July 25, 2011. These results indicate that eggplant under relay intercropping with normal garlic or green garlic suffered less damage from environment or continuous cropping obstacles than monocropped eggplant.

### Effect of Relay Intercropping with Normal Garlic or Green Garlic on SOD Activity in Eggplant Leaves

**FIGURE 2:**
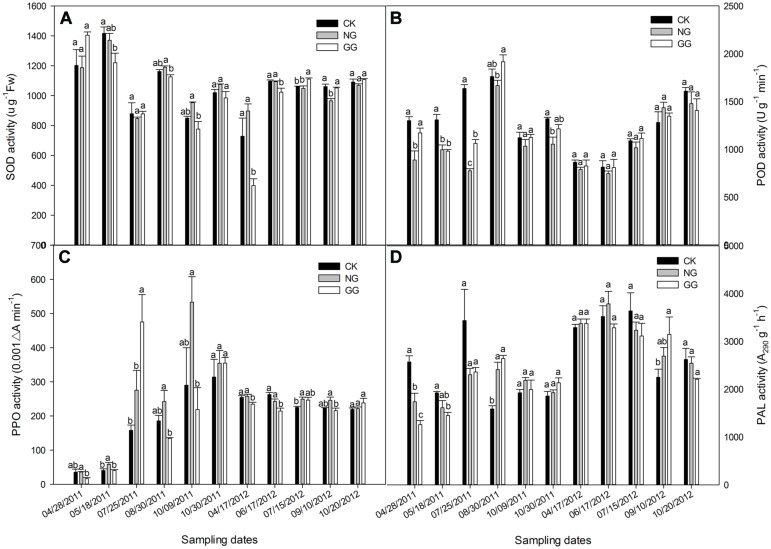
**Effects of relay intercropping with normal garlic or green garlic on superoxide dismutase (SOD) **(A)**, peroxidase (POD) **(B)**, polyphenol oxidase (PPO) **(C)** and phenylalanine ammonia-lyase (PAL) **(D)** activities in eggplant leaves.** Error bars represent the SE of the mean. The letters a, b, and c indicate significant differences at the *p* < 0.05 level (ANOVA and LSD), and *n* = 3. CK, eggplant monoculture; NG, eggplant under relay intercropping with normal garlic; GG, eggplant under relay intercropping with green garlic.

The SOD activity in the eggplant leaf in 2011 and 2012 is shown in **Figure 2A**. In 2011, the SOD activity followed an M-shaped variation with an overall downward trend; in 2012, it was lowest in the April 17 samples and slowly increased after that date. In 2011, the SOD activity in the GG treatment was lower than that in the CK treatment, except on April 28, and the difference reached significance on May 18. Relay intercropping with green garlic may protect the eggplant plants against damage from the external environment, thus reducing the need to SOD activity. The SOD activity in the NG treatment was lower than that in the CK treatment before August but was higher after August 30; however, the difference between the NG and CK treatments over the entire year was not significant. For two sampling dates in 2011 — August 30 and October 9 — the SOD activity in the GG treatment was significantly lower than that in the NG treatment. More garlic was present in the GG treatment (∼8.5 kg) than in the NG treatment (∼0.35 kg); therefore, green garlic played a more important role than normal garlic in protecting the eggplant plants. In 2012, the SOD activity in the GG treatment was lower than that in the CK treatment for most sampling dates, with some dates showing significant differences. In general, the SOD activity in the relay intercropping treatments in 2012 was lower than that in the monoculture treatment. The lower SOD activity in the NG and GG treatments indicates that relay intercropping with normal garlic or green garlic could offer better protection for eggplant plants compared with monoculture-grown plants.

### Effect of Relay Intercropping with Normal Garlic or Green Garlic on POD Activity in Eggplant Leaves

As shown in **Figure 2B**, the POD activity in 2011 initially increased and then decreased, whereas in 2012, it overall continued to increase. In 2011, the POD activity in the NG treatment was lower than that in the CK treatment for all sampling dates, and most of the differences were significant. The POD activities in the GG treatment were similar to those in the NG treatment, also lower than that in the CK, except for the sample collected on August 30. However, the POD activity in GG was not significantly different from that in CK for most of the sampling dates. The POD activity in all treatments reached maximum values during the fruit-bearing stage, when the temperature was high, indicating that eggplant plants may suffer a certain degree of damage under high temperatures. On October 9, the POD activity fell sharply with the decrease in temperature and then increased slightly on October 30, indicating that the eggplant plants reached the period of consenescence. In 2012, the variation trend was quite different from that in 2011. Different climate conditions in the different continuous cropping years may have caused the different growth characteristics and POD trends. Although the POD activities in the NG and GG treatments were lower than that in CK for most sampling dates in 2012, no significant differences were recorded.

### Effect of Relay Intercropping with Normal Garlic or Green Garlic on PPO Activity in Eggplant Leaves

The PPO activity for the 2 years is shown in **Figure 2C**. In 2011, the PPO activity continually increased in the CK treatment from the seedling phase to the senescence phase. In the NG and GG treatments, however, irregular M-shaped curves were observed. The PPO activity in the NG treatment was higher than that in the CK treatment, but only on May 18, the difference between them was significant, whereas the value in the GG treatment was significantly lower than that in the CK treatment in most sampling dates except for July 25 and October 30, 2011. In 2012, the PPO activity generally followed a slow decline, but there were no significant differences between the CK and NG treatments for most of the sampling dates except that on July 15, 2012, the PPO activity in the NG was significantly higher than that in CK. In the GG treatment, the PPO activity was often lower than that in the CK treatment. In both years, the PPO activity in the GG treatment exceeded that in the CK treatment 5 days before the green garlic was planted (July 15, 2011 and July 25, 2012) and then decreased after the green garlic took root (August 30, 2011 and September 10, 2012). This result was likely due to effects resulting from the growth of the garlic roots and its exudates.

### Effect of Relay Intercropping with Normal Garlic or Green Garlic on PAL Activity in Eggplant Leaves

The overall trend for PAL activity was similar to that for the MDA content (**Figure 2D**). On the first three sampling dates in 2011, which was from the eggplant seedling stage to fruiting period, the PAL activity in the CK treatment was higher than that in either the NG or GG treatments. However, the opposite trend was observed over the next three sampling dates when the eggplant gradually entered the senescence stage. In 2012, although the PAL activity in the CK treatment was sometimes higher or lower than that in the other two treatments, there were no significant differences among the three treatments, except for the significantly higher PAL activity in the GG treatment on September 10 compared to that in the CK treatment. However, in both years, the PAL activity in the GG treatment was lower than that in the CK treatment 5 days before the green garlic was planted and then increased and was higher in the GG treatment than in CK, which was opposite to the PPO. At the full-bearing stage of eggplant, the PAL activity reached its highest level during the most vigorous growth period. This increased PAL activity may protect eggplant plants from the various external stresses caused by continuous cropping or unsuitable climate conditions.

### Effect of Relay Intercropping with Normal Garlic or Green Garlic on Eggplant Growth and Yield

**FIGURE 3:**
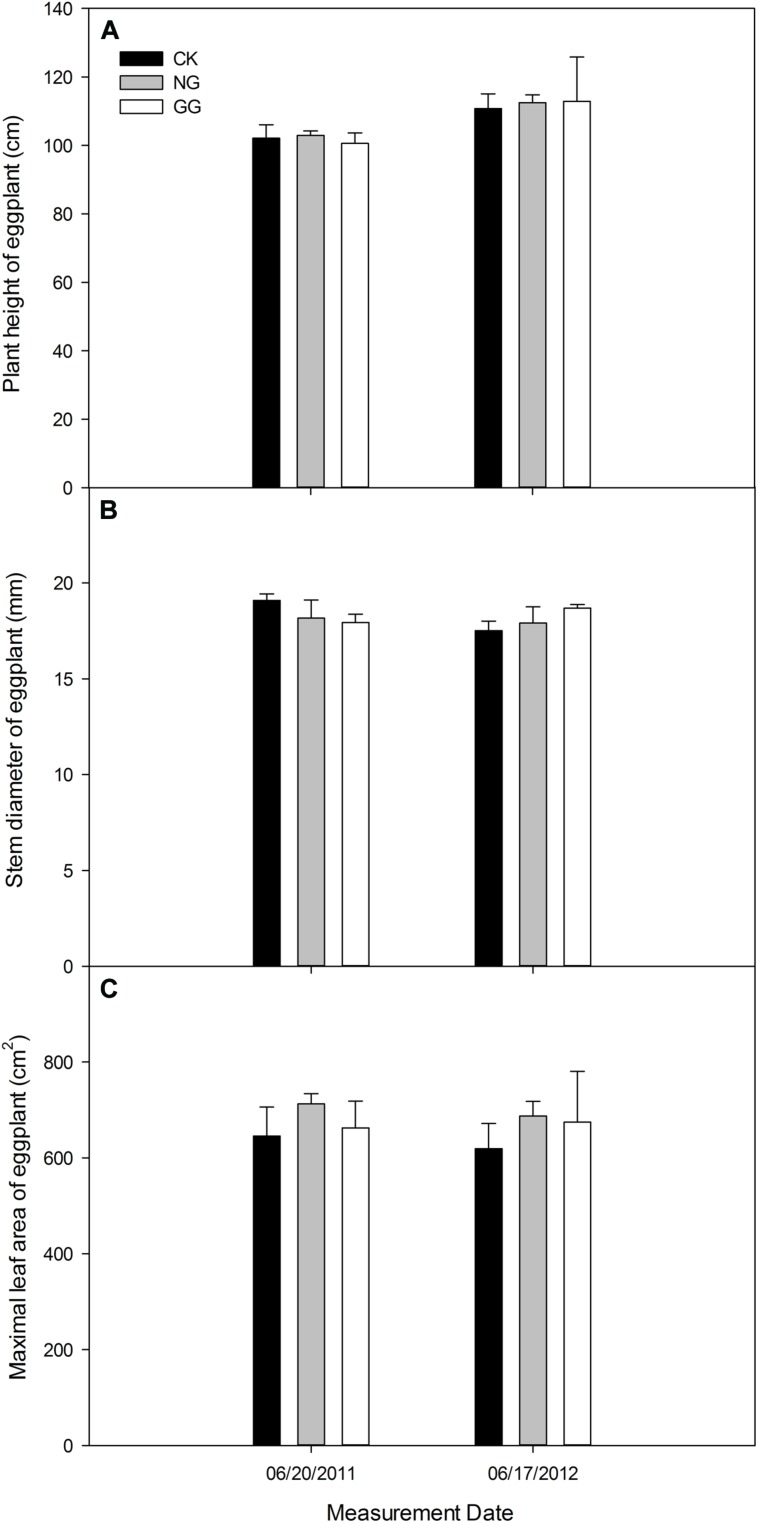
**Effects of relay intercropping with normal garlic or green garlic on the plant height **(A)**, stem diameter **(B)** and maximum leaf area **(C)** of eggplant.** Error bars represent the standard error of the mean, *n* = 3. CK, eggplant monoculture; NG, eggplant under relay intercropping with normal garlic; GG, eggplant under relay intercropping with green garlic.

On June 20, 2011, the eggplant in NG treatment was a little higher than CK, but it was lower in GG than CK. On June 17, 2012, the plant in NG and GG treatments was both higher than CK (**Figure 3A**). For the stem diameter in **Figure 3B**, the eggplant was thinner in NG and GG treatments than CK in 2011, but thicker in 2012. The maximal leaf area (**Figure 3C**) was larger in NG and GG treatments than CK at eggplant vigorous growth stage both in 2011 and 2012.

As shown in **Table 1**, in 2011, the eggplant yield in NG treatment was 2.85% higher in NG than CK, but 18.40% lower in GG. Then after moving the time of pulling out green garlic forward in 2012, the yields of both NG and GG were higher than CK, respectively, increased by 6.26 and 7.80%.

**Table 1:** Eggplant yields of the three treatments in 2011 and 2012.

Treatment	Yield (kg⋅ha^-1^)	
	2011	2012
CK	99611 ± 2709	71141 ± 4610
NG	102448 ± 2601	75595 ± 5554
GG	81282 ± 5343	76692 ± 8338

*CK, eggplant monoculture; NG, eggplant under relay intercropping with normal garlic; GG, eggplant under relay intercropping with green garlic.*

*Data are presented as means ± SE, *n* = 3.*

## Discussion

Plants suffer from many types of environmental stresses over the course of growth and development, including extreme temperatures (high temperature or low temperature) and strong daylight conditions. Continuous monoculture cultivation also results in serious stress to plants, such as weed invasion and pest and disease infestation (Blanco and Lal, 2010). These physiological stresses can result in rapidly increasing amounts of ROS in plant cells, damaging the cellular structure and decreasing the nutritional quality of their fruit and vegetative parts (Hagerman et al., 2003; Gechev et al., 2006; Möller and Sweetlove, 2010; Singh et al., 2012; Oukarroum et al., 2015). Membrane lipid peroxidation of plant tissues often occurs in abiotic and senescence stresses (Chen et al., 2011a). As one of the products of membrane lipid peroxidation, MDA is correlated to the degree of membrane lipid peroxidation (Adams et al., 2008; Wu et al., 2014). The MDA content in eggplant plants relay intercropped with both normal and green garlic was lower than that in monocropped plants, indicating that relay intercropping with garlic protected the plants from damage. Moreover, for all three treatments, the MDA content reached its maximum level during the fruit-bearing period from June to August, a time when temperatures were at their highest. During this period, temperatures may exceed 50°C in the plastic tunnel (Wang et al., 2014); however, the physiological limit of eggplant is 35°C, and higher temperatures cause the crop to grow slowly and develop poorly. The injuries suffered by eggplants due to high temperatures are thought to increase the MDA content. Nevertheless, the significantly lower MDA content in the relay intercropping systems shows that relay intercropping with garlic can reduce the damage suffered by eggplant and that relay intercropping can improve the plants’ resistance to high temperature or other stress.

It is known that environmental stresses often induce activity for plants free-radical detoxification enzymes such as SOD and POD (Shah et al., 2001; Nahakpam and Shah, 2011; Liu and Wang, 2012; Rai et al., 2012). The producing and scavenging of ROS in cells is always in a dynamic balance, as is the activity of the enzymes responsible. SOD is the first enzyme involved in the detoxifying process that converts O_2_^⋅-^ radicals to H_2_O_2_ at a very rapid rate (Hasan et al., 2009). During the eggplant growth stages, the SOD level varied as an irregular M-shape in 2011. However, the SOD activity in the NG and GG treatments was lower than that in the CK treatment for most sampling dates, showing that relay intercropping with garlic can prevent the plants from being injured by pests, pathogens, or other complications of continuous cropping. There are several possible mechanisms for this phenomenon: first, garlic volatile substances or root exudates may have inhibited pests and pathogens (Wei et al., 2011); after the garlic was removed from the field, its residual root exudates may have remained, acting as allelochemicals on soil microorganisms, especially soil-borne pathogens (Khan et al., 2011). In addition, there were two sampling dates on which the SOD activity in the GG treatment was significantly lower than that in the NG treatment. The two different cultivation patterns, with different garlic densities, produced different amounts of allelochemicals, and presumably led to different protection levels, such as at the level of the spatial isolation of pests (Lithourgidis et al., 2011) or conidia (Gómez-Rodríguez et al., 2003), microbial antagonism (Gómez-Rodríguez et al., 2003; Khan and Cheng, 2010), or allelopathy from different volumes of root exudates (Khan et al., 2011), as reported in similar studies.

Induction of POD activity has been documented under many stress conditions such as salt stress (Chen et al., 2011a), pest damage (Dowd and Lagrimini, 2006), or pathogen injection (Melgar et al., 2006). POD is one of the most important enzymes involved in regulation of intracellular level of H_2_O_2_ (Hiraga et al., 2001; Passardi et al., 2005). When the plants suffer damage, more $O_{2}^{∙-}$ would produce, and SOD could dismutate these $O_{2}^{∙-}$; subsequently, excessive H_2_O_2_ induce the over expression of POD gene (Li et al., 2013). Therefore, both the SOD and POD activities would increase to avoid injury to plant cells. In this experiment, the overall trend of the SOD and POD activities were similar. In 2011, the trends were irregularly M-shaped, and in 2012, both reached their lowest values on 17 April, followed by a general increase. Moreover, as the last catalytic step in the polymerization of lignin, POD provides an important defense against the intrusion and extension of pathogens (Johrde and Schweizer, 2008; Weissinger et al., 2013). Our results showed that the POD activity in eggplant plants relay intercropped with garlic was lower than that in CK. Indeed, relay intercropping systems are known to result in less disease or other damage than monoculture systems (Suman et al., 2000; Cai et al., 2010).

POD and PPO act synergistically in enzymatic browning because PPO can promote POD activity by generating H_2_O_2_ from the oxidation of phenolic compounds (Tomás-Barberán and Espín, 2001; Criado et al., 2015). These enzymes play an important role in plant defense through the oxidation of endogenous phenolic compounds into quinones, which are toxic to invading pathogens and pests (Dowd et al., 2000). The resulting quinones may undergo non-enzymatic autopolymerization or covalent heterocondensation with proteins and carbohydrates to produce colored compounds (Tomás-Barberán and Espín, 2001), and these compounds may constitute a barrier against biotic and abiotic stresses (Abdel-Aal et al., 2001). Furthermore, it has been suggested that POD inhibits PPO activity (Shinshi and Noguchi, 1975). Therefore, in general, when the POD activity was higher, the PPO activity of the same sampling dates was lower. However, several complicating factors led to somewhat variable results. On June 17, 2011, both the POD and PPO activities of the CK treatment were higher than that of NG and GG treatments. As Mohammadi and Kazemi (2002) found that, the POD and PPO activities in wheat heads were increased considerably following infection by *Fusarium graminearum*.

Previous studies have found that additional defense systems are activated when plants suffer from damage, especially the phenylpropanoid metabolism system, and PAL activity increases rapidly (Dixon et al., 2002; Ge et al., 2014; Talbi et al., 2015). Therefore, PAL activity is an important physical indicator of a plant’s ability to resist adversity. Diaz et al. (2007) found that PAL activity was significantly reduced by selenium (Se) treatments in *Lactuca sativa* at harvest time. Se is well known for its high potential to protect plant membranes, eradicate free particles, and delay senescence (Djanaguiraman et al., 2004; Diao et al., 2014; Hajiboland and Sadeghzade, 2014). The same theory proves that in our study, the lower PAL activity in the relay intercropping treatments showed that relay intercropping with garlic may protect eggplant biomembranes or delay senescence.

However, researchers found that PAL activity was higher in the lignified tissue of many varieties of plants, though PAL activity was not detected in the unlignified tissues of the same plants (Moller et al., 2006). In addition, some studies showed that PAL can catalyze the synthesis of anthocyanidin (Fischer et al., 2007; Yu et al., 2012), which is an important component of the color of flowers, fruits, and leaves. In this experiment, PAL activity in the CK treatment was higher than that in the NG and GG treatments in the first half of 2011. This difference may have been caused by the garlic in the field shielding the small eggplant seedlings from sunlight, thereby blocking photosynthesis and competing with the eggplant plants for nutrition, which may have caused reduced lignification and weaker eggplant growth in the relay intercropping treatments, ultimately leading to the lower yield in the GG treatment. The higher soil nutrition in NG and GG treatments in the corresponding sampling dates (Wang et al., 2014) could not be enough to save these adverse impacts. Increases in the synthesis of certain substances such as anthocyanin, is important for eggplant growth and fruit coloring, accompanies fruit development and the degree of lignification also increases, leading to increase in PAL activity. Jouili and Ferjani (2003) found that the activity of PAL, which plays an important role in plant defense, was activated under cupric stress conditions. This phenomenon occurred in the relay intercropping periods in 2012. However, the opposite trend was observed when the eggplant gradually entered the senescence stage, which might be because in the same period, the senescence of the eggplant in NG and GG treatments was slower than that in CK, as Subrarnaniam et al. (1993) found that, the PAL activity was higher in juvenile leaf, terminal bud and caulicle of poplar, but lower in older stems and mature leaves. In addition, the parabola-like curve of PAL activity may indicate that the eggplant plants were damaged by high temperatures during the hottest days of the year, inducing increases in PAL activity to prevent injury, which is consistent with the study results of Wang (2011).

Many studies have shown that the activities of such stress resistance-related enzymes are low in healthy plants (Panina et al., 2005; Zhang et al., 2008; Hashempour et al., 2014). It reflected conversely on the crop growth and yield in 2012 that eggplant was stronger and the yield was higher in relay intercropping treatments than that in monoculture treatment, indicating that relay intercropping with garlic has stimulative effect to eggplant growth and yield. The higher activities of enzymes and MDA content observed in the eggplant leaves might indicate more injuries suffered in monoculture system, leading to weaker growth condition and lower yield, whereas healthier plant in relay intercropping systems led to stronger growth and higher yield. Furthermore, the higher soil enzyme activities and nutrients in eggplant/garlic relay intercropping systems (Wang et al., 2014) may also lead to healthier plant, and then lower plant enzyme activities and higher yield. Although in 2011, the eggplant yield in GG treatment was lower than that in CK because of the sunlight blocking effect of thick green garlic on the eggplant seedlings; however, after moving the time of pulling out green garlic forward in 2012, the yield in GG treatment was higher than that in CK. Although monocropping systems cause biological stress with a continuous target over time and space, intercropping systems can better control such injury by promoting biological diversity, keeping healthy plant growth and reducing the risk of crop losses.

## Conclusion

The relay intercropping of eggplant with normal or green garlic is a beneficial production practice because it can alleviate the injuries to eggplant resulting from continuous cropping obstacles to the main crop and maintaining the stronger plant growth and higher yield, which provides further verification of the advantages of relay intercropping systems. Therefore, eggplant/garlic relay intercropping systems can facilitate the sustainable development of eggplant production. Furthermore, these results provide convincing evidence of the efficacy of relay intercropping that may be useful in similar systems. There were some discrepant results observed in different parameters, because the plant could be affected by many biotic and abiotic factors during its different growth periods. To more deeply understand the protection mechanism of those plant enzymes, gene expression will be done in our further study.

## Author Contributions

All authors made contributions to the experiment and manuscript. MW made substantial contributions to the design of the work, the acquisition, analysis, and interpretation of data for the work and drafting the work. CW made contributions to the acquisition and analysis of date for the work and revising it critically for important intellectual content. ZC made contributions to the conception of the work and revising it critically for important intellectual content. HM made contributions to the acquisition of data for the work and revising the manuscript. All authors made contributions to the final approval of the version to be published and agreement to be accountable for all aspects of the work in ensuring that questions related to the accuracy or integrity of any part of the work are appropriately investigated and resolved.

## Conflict of Interest Statement

The authors declare that the research was conducted in the absence of any commercial or financial relationships that could be construed as a potential conflict of interest.

## Abbreviations

CAT: catalase
CK: eggplant monoculture
GG: eggplant relay intercropping with green garlic
MDA: malondialdehyde
NBT: nitro blue tetrazolium
NG: eggplant relay intercropping with normal garlic
PAL: phenylalanine ammonia-lyase
POD: peroxidase
PPO: polyphenol oxidase
ROS: reactive oxygen species
SOD: superoxide dismutase
TBA: thiobarbituric acid
TCA: trichloroacetic acid.
